# Stability analysis of nonlinear localized modes in the coupled Gross-Pitaevskii equations with PT -symmetric Scarf-II potential

**DOI:** 10.1371/journal.pone.0294790

**Published:** 2023-11-27

**Authors:** Jia-Rui Zhang, Xia Wang

**Affiliations:** College of Science, China Agricultural University, Beijing, China; Tel Aviv University, ISRAEL

## Abstract

We study the nonlinear localized modes in two-component Bose-Einstein condensates with parity-time-symmetric Scarf-II potential, which can be described by the coupled Gross-Pitaevskii equations. Firstly, we investigate the linear stability of the nonlinear modes in the focusing and defocusing cases, and get the stable and unstable domains of nonlinear localized modes. Then we validate the results by evolving them with 5% perturbations as an initial condition. Finally, we get stable solitons by considering excitations of the soliton via adiabatical change of system parameters. These findings of nonlinear modes can be potentially applied to physical experiments of matter waves in Bose-Einstein condensates.

## 1 Introduction

Bose-Einstein condensate (BEC) [1, 2], as one of the important physical phenomena, has attracted the attention of researchers. The successful observation of solitons in BECs has become one of the research focuses in the fields of condensed matter physics and atom optics [3–5]. Compared with the single-component ones, the multi-component BECs possess the inter-component interactions and have complicated quantum phases and properties [6–13]. Many novel phenomena have been discovered in multi-component BECs [14–22], including symbiotic solitons, soliton trains, soliton pairs, multi-domain walls, and multi-mode collective excitations. As one kind of multi-component BECs, the two-component BECs trapped in a quasi-one-dimensional harmonic potential at zero temperature can be described by the following coupled Gross-Pitaevskii equations [23, 24]:

$$\begin{array}{c} i\hbar \frac{\partial \Psi_{1}}{\partial t}=(-\frac{\hbar^{2}}{2M}\nabla^{2}+V_{1}\left(r\right)+g_{11}|\Psi_{1}{|}^{2}+g_{12}{\left|\Psi_{2}\right|}^{2})\Psi_{1},\; \end{array}$$ (1a)

$$\begin{array}{c} i\hbar \frac{\partial \Psi_{2}}{\partial t}=(-\frac{\hbar^{2}}{2M}\nabla^{2}+V_{2}\left(r\right)+g_{21}|\Psi_{1}{|}^{2}+g_{22}{\left|\Psi_{2}\right|}^{2})\Psi_{2},\; \end{array}$$ (1b)

where Ψ_*j*_ is the two-component parameter, ***r*** = (*x*, *y*, *z*), ℏ is the Planck constant, *M* is the atomic mass, ∇^2^ is the Laplacian, $V_{j}\left(r\right)=\left[\omega_{jx}^{2}x^{2}/2+\omega_{j⊥}^{2}\left(y^{2}+z^{2}\right)\right]M/2$ stands for the harmonic potentials, *g*_*jj*_ is the interactions between atoms, and *g*_*j*,3−*j*_ describes the inter-component interactions (*j* = 1, 2) [25–28].

If the trap frequencies in the radial directions *ω*_*j*⊥_ are larger than the axial directions *ω*_*jx*_, Eq (1) becomes a quasi-one-dimensional system along the *x* direction. Through the normalization and transformation Ψ_*j*_ → *ψ*_*j*_, $x\to \sqrt{\hbar /(M\omega_{1⊥})}x$, *t* → (2*π*/*ω*_1⊥_)*t*, Eq (1) can be written in the form [25–28]:

$$\begin{array}{c} i\frac{\partial \psi_{1}}{\partial t}=(-\frac{1}{2}\frac{\partial^{2}}{\partial x^{2}}+\frac{\lambda_{1}^{2}}{2}x^{2}+b_{11}|\psi_{1}{|}^{2}+b_{12}{\left|\psi_{2}\right|}^{2})\psi_{1},\; \end{array}$$ (2a)

$$\begin{array}{c} i\frac{\partial \psi_{2}}{\partial t}=(-\frac{1}{2}\frac{\partial^{2}}{\partial x^{2}}+\frac{\lambda_{2}^{2}}{2}x^{2}+b_{21}|\psi_{1}{|}^{2}+b_{22}{\left|\psi_{2}\right|}^{2})\psi_{2},\; \end{array}$$ (2b)

where λ_*j*_ = *ω*_*jx*_/*ω*_*j*⊥_, *b*_*jj*_ and *b*_*j*,3−*j*_ are related to $\int |\psi_{j}{|}^{2}\underset{˙}{\text{x}}$, trap frequencies in the radial directions *ω*_*j*⊥_, and interactions between atoms *g*_*jj*_ or inter-component *g*_*j*,3−*j*_ (*j* = 1, 2). For the harmonic potentials, the soliton states were studied widely [25–28]. In this paper, we consider the two-component BECs trapped in another potential.

Put forward by Bender and his coworker in 1998 [29–31], parity-time- (PT-) symmetry behaviors have attracted much attention in both non-Hermitian Hamiltonian systems and nonlinear wave systems [32–34], which make systems with complex potentials possibly support fully-real linear spectra [35] and stable nonlinear modes [36–39]. That is, the potential function *U*(*x*) = *V*(*x*) + *iW*(*x*) satisfies *V*(*x*) = *V*(−*x*) and *W*(−*x*) = −*W*(*x*) [29–31]. Over the past few years, various PT-symmetric potentials have been introduced into the nonlinear Schrödinger equation and the existence of different nonlinear local modes is analytically and numerically investigated [40–51]. To better investigate physical phenomena, it is meaningful to introduce new forms of PT-symmetric potentials in nonlinear systems.

In this paper, we investigate the coupled Gross-Pitaevskii equations with complex PT-symmetric potentials [28]:

$$\begin{array}{c} i\frac{\partial \psi_{1}}{\partial t}=(-\frac{\partial^{2}}{\partial x^{2}}-a_{1}\left(\right|\psi_{1}{|}^{2}+|\psi_{2}{|}^{2})-U_{1}\left(x\right))\psi_{1},\; \end{array}$$ (3a)

$$\begin{array}{c} i\frac{\partial \psi_{2}}{\partial t}=(-\frac{\partial^{2}}{\partial x^{2}}-a_{2}\left(\right|\psi_{1}{|}^{2}+|\psi_{2}{|}^{2})-U_{2}\left(x\right))\psi_{2},\; \end{array}$$ (3b)

where *a*_*j*_ represent the intra-component and inter-component interactions while the interactions take equal values when *a*_1_ = *a*_2_, *U*_*j*_(*x*) are the complex PT-symmetric potentials, and the imaginary parts of *U*_*j*_(*x*) stand for the gain or loss term from the thermal clouds (*j* = 1, 2).

The present paper is built up as follows. In Sect. 2, we consider the analytic bright-soliton solution in the coupled Gross-Pitaevskii equations with complex PT-symmetric Scarf-II potentials; the linear stability analysis and the numerical evolution results corroborating the analytical solitons are presented in Sect. 3; In Sect. 4, we perform numerical simulations for the excitation and evolution of nonlinear modes via adiabatical change of system parameters; Finally, conclusions and discussions are given in Sect. 5.

## 2 Localized modes in coupled Gross-Pitaevskii equations

We concentrate on stationary solutions of Eq (3) in the form

$$\begin{array}{c} \psi_{j}\left(x,t\right)=\phi_{j}\left(x\right){\text{e}}^{i\nu_{j}t},\;j=1,2\;, \end{array}$$ (4)

where *ν*_*j*_ are the real propagation constant. The complex solutions *ϕ*_*j*_(*x*) satisfy the following condition

$$\begin{array}{c} {}[\frac{d^{2}}{dx^{2}}+a_{1}\left(\right|\phi_{1}{|}^{2}+|\phi_{2}{|}^{2})+U_{1}\left(x\right)]\phi_{1}=\nu_{1}\phi_{1}\;, \end{array}$$ (5a)

$$\begin{array}{c} {}[\frac{d^{2}}{dx^{2}}+a_{2}\left(\right|\phi_{1}{|}^{2}+|\phi_{2}{|}^{2})+U_{2}\left(x\right)]\phi_{2}=\nu_{2}\phi_{2}\;, \end{array}$$ (5b)

which can be solved for the given potentials *U*_*j*_(*x*) and real propagation constant *ν*_*j*_.

For the PT-symmetric potentials *U*_*j*_(*x*) are all chosen as the well-known Scarf-II potentials as

$$\begin{array}{c} U_{j}\left(x\right)=V_{j}\;{\text{sech}}^{2}\left(x\right)+iW_{j}\;\text{sech}\left(x\right)\;\text{tanh}\left(x\right). \end{array}$$ (6)

We have the analytic bright-soliton solution as [37]

$$\begin{array}{c} \phi_{j}\left(x\right)=A_{j}\;\text{sech}\left(x\right)\;{\text{e}}^{i\varphi_{j}},\;j=1,2\;, \end{array}$$ (7)

with the phases being

$$\begin{array}{c} \phi_{j}=\frac{W_{j}}{3}\;\text{arctan}\left[\text{sinh}\left(x\right)\right],\;j=1,2\;, \end{array}$$ (8)

under the constraints of

$$\begin{array}{c} \frac{18+W_{j}^{2}-9V_{j}}{9a_{j}}=\sum_{n=1,2}A_{n}^{2},\;j=1,2\;, \end{array}$$ (9)

and *ν*_*j*_ = 1.

For the nonlinear modes given in Eq (7), the power of the solutions is defined as $P_{j}=\int_{-\infty }^{\infty }{\left|\phi_{j}\left(x\right)\right|}^{2}dx$, *P* = *P*_1_ + *P*_2_, while the Poynting vector $S_{j}=\frac{i}{2}\left(\phi_{j}\phi_{jx}^{*}-\phi_{j}^{*}\phi_{jx}\right)=A_{j}^{2}W_{j}/3\;{\text{sech}}^{3}\left(x\right)$. The power flows from left (right) to right (left) at *x*_0_ when *S*(*x*_0_) > 0 (*S*(*x*_0_) < 0).

## 3 Linear stability analysis

In this section, we investigate the linear stability of the nonlinear modes, which is a standard protocol to show the stability of nonlinear localized modes. We consider the perturbed solution *ψ*_*j*_(*x*, *t*), in the form

$$\begin{array}{c} \psi_{j}\left(x,t\right)=\phi_{j}\left(x\right)\;{\text{e}}^{i\nu_{j}t}+\epsilon \;[f_{j}\left(x\right)\;{\text{e}}^{i\delta t}+g_{j}^{*}\left(x\right)\;{\text{e}}^{-i\delta^{*}t}]\;{\text{e}}^{i\nu_{j}t}\; \end{array}$$ (10)

where *ϵ* ≪ 1, which is the small perturbation on the solution. *f*_*j*_(*x*) and *g*_*j*_(*x*) are the perturbation eigenfunctions of the linearized eigenvalue problem. By substituting Eq (10) into Eq (5) and linearizing with respect to *ϵ*, we can drive the following linear eigenvalue problem:

$$\begin{array}{c} (\begin{array}{cccc} L_{1} & a_{1}\phi_{1}^{2} & a_{1}\phi_{1}\phi_{2}^{*} & a_{1}\phi_{1}\phi_{2}\\ -a_{1}\phi_{1}^{*2} & -L_{1}^{*} & -a_{1}\phi_{1}^{*}\phi_{2}^{*} & -a_{1}\phi_{1}^{*}\phi_{2}\\ a_{2}\phi_{1}^{*}\phi_{2} & a_{2}\phi_{1}\phi_{2} & L_{2} & a_{2}\phi_{2}^{2}\\ -a_{2}\phi_{1}^{*}\phi_{2}^{*} & -a_{2}\phi_{1}\phi_{2}^{*} & -a_{2}\phi_{2}^{*2} & -L_{2}^{*} \end{array})(\begin{array}{c} f_{1}\\ g_{1}\\ f_{2}\\ g_{2} \end{array})=\delta (\begin{array}{c} f_{1}\\ g_{1}\\ f_{2}\\ g_{2} \end{array}), \end{array}$$ (11)

where

$$\begin{array}{c} L_{1}=-\nu_{1}+\partial_{x}^{2}+U_{1}+2a_{1}\phi_{1}\phi_{1}^{*}+a_{1}\phi_{2}\phi_{2}^{*}\; \end{array}$$ (12a)

$$\begin{array}{c} L_{2}=-\nu_{2}+\partial_{x}^{2}+U_{2}+a_{2}\phi_{1}\phi_{1}^{*}+2a_{2}\phi_{2}\phi_{2}^{*}\; \end{array}$$ (12b)

The imaginary part of *δ* measures the growth rate of the perturbation instability. If |Im(*δ*)| > 0, then the perturbation will grow exponentially with *t*, and the solutions are unstable; otherwise, the solutions are stable. In our numerical simulation, we use the Fourier collocation method to discretize the associated differential operator as a matrix to solve the eigenvalue problem [52]. To further verify the stability of the solitons, we numerically investigate the stability by evolving them with 5% perturbations as the initial condition to simulate the random white noise (i.e., *ψ*(*x*, 0) = *ϕ*(*x*)(1 + *ξ*) and *ξ* represents 5% perturbations). In our numerical simulations, the second-order spatial differential is carried out by using Fourier spectral collocation method, and the integration in time is carried out by using the explicit fourth-order Runge-Kutta method [53].

**Fig 1:**
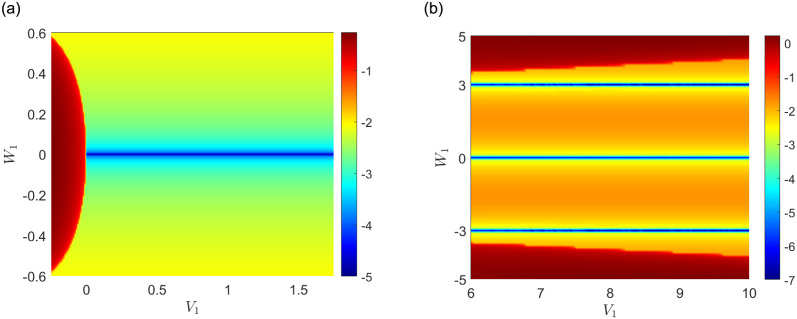
Maximal imaginary part of the linearization eigenvalue *δ* in the (*V*_1_, *W*_1_)-space (common logarithmic scale), under the constraint of *A*_1_ = 0.5, *V*_1_ = *V*_2_, *W*_1_ = *W*_2_ and (a) *a*_1_ = 1; (b) *a*_1_ = −1.

Firstly, under the constraint of *A*_1_ = 0.5, *V*_1_ = *V*_2_, *W*_1_ = *W*_2_, we consider the focusing case *a*_1_ = *a*_2_ = 1 and the defocusing case *a*_1_ = *a*_2_ = −1, respectively. Then we get the stable (blue) and unstable (red) domains of nonlinear localized modes in (*V*_1_, *W*_1_) space [see Fig 1]. They are determined by the maximum absolute value of imaginary parts of the linearized eigenvalue *δ* in Eq (11). We find that solitons tend to be unstable with the increase of |*W*_1_| in the focusing case. It is worth noting that when |*W*_1_| = 3, solitons are stable in the defocusing case.

**Fig 2:**
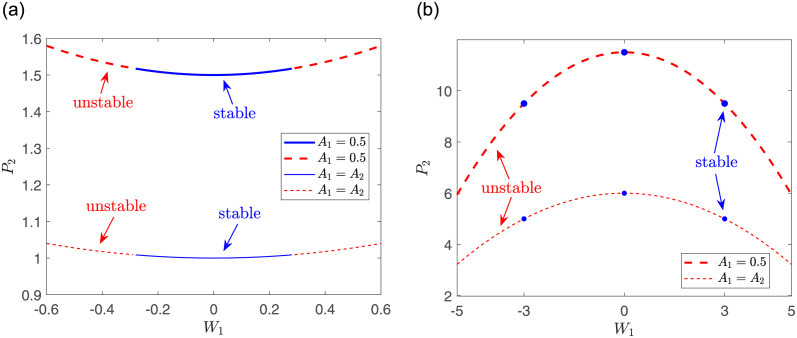
The relationship between power of nonlinear mode *ϕ*_2_ and *W*_1_. The parameters are chosen as: *W*_1_ = *W*_2_, and (a) *V*_1_ = *V*_2_ = 1, *a*_1_ = 1; (b) *V*_1_ = *V*_2_ = 8, *a*_1_ = −1.

Since the above situations are obtained in the case of *A*_1_ = 0.5, and *A*_2_ is obtained by Eq 9. Next, we consider the case of *A*_1_ = *A*_2_. The relationships between *P*_2_ and the parameter *W*_1_ are shown in Fig 2. We can find that when other parameters are fixed, *P*_2_ and |*W*_1_| are positively correlated in the focusing case, while they are negatively correlated in the defocusing case. In addition, for both cases *A*_1_ = 0.5 and *A*_1_ = *A*_2_, the intervals of *W*_1_ have no difference when the solutions are stable.

**Fig 3:**
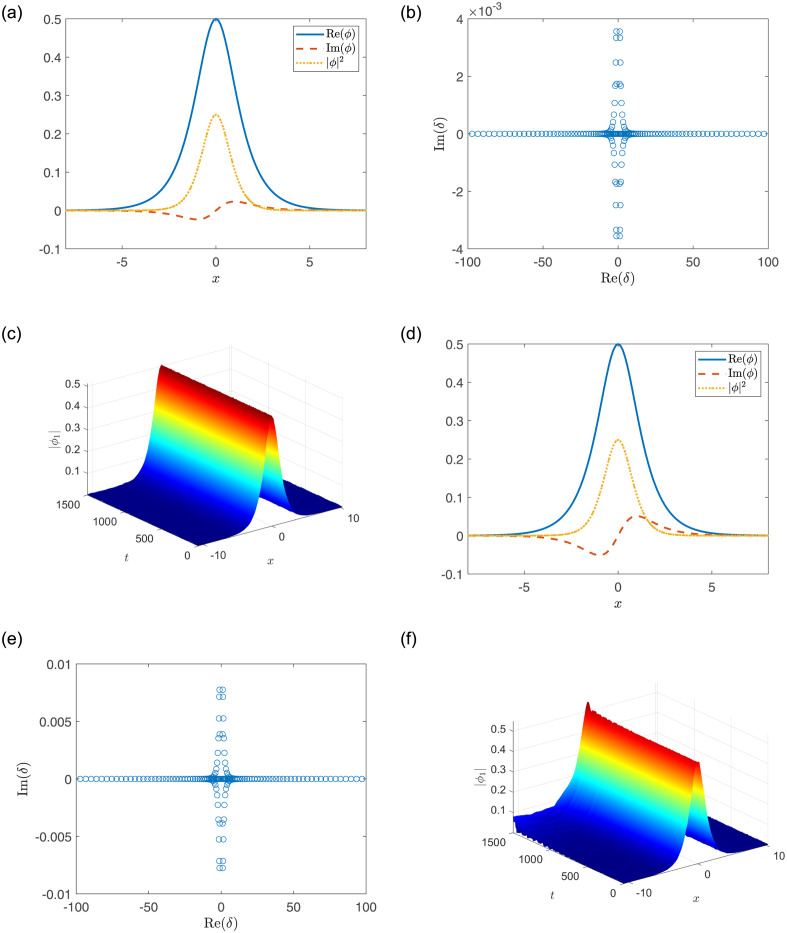
(a, d) The soliton solutions. (b, e) Linear stability eigenvalues. (c, f) Stable or unstable propagations of nonlinear modes. The parameters are chosen as: *a*_1_ = 1, *A*_1_ = 0.5, *V*_1_ = *V*_2_ = 1, and (a-c) *W*_1_ = *W*_2_ = 0.25; (d-f) *W*_1_ = *W*_2_ = 0.55.

**Fig 4:**
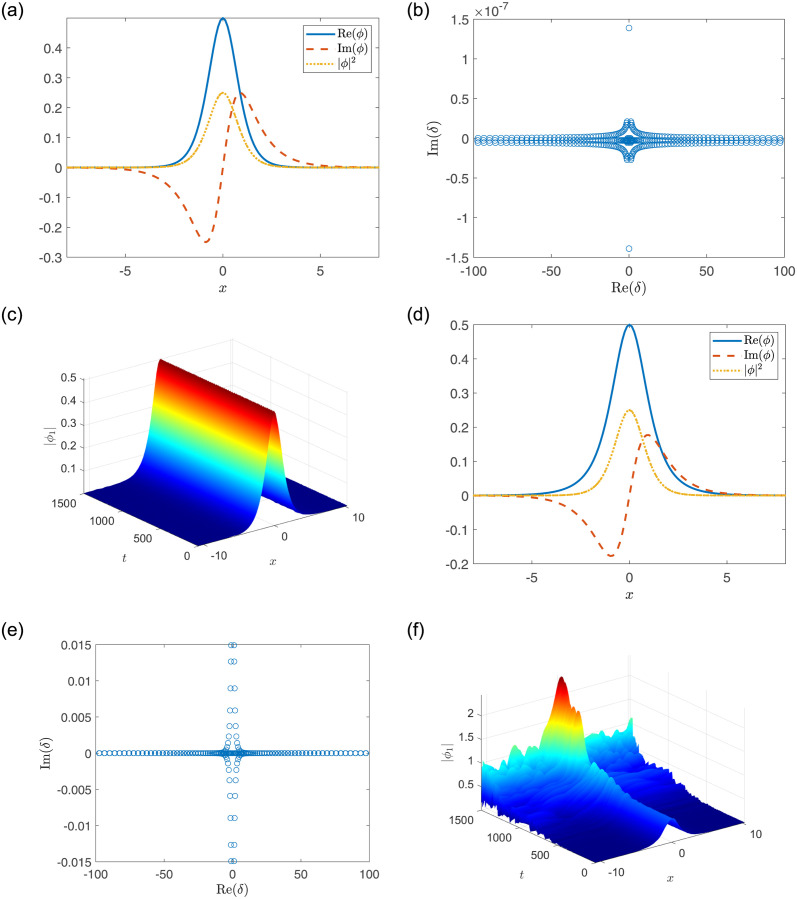
(a, d) The soliton solutions. (b, e) Linear stability eigenvalues. (c, f) Stable or unstable propagations of nonlinear modes. The parameters are chosen as: *a*_1_ = −1, *A*_1_ = 0.5, *V*_1_ = *V*_2_ = 8, and (a-c) *W*_1_ = *W*_2_ = 3; (d-f) *W*_1_ = *W*_2_ = 2.

In particular, for the fixed parameters *a*_1_ = *a*_2_ = 1, *A*_1_ = 0.5, *V*_1_ = *V*_2_ = 1, Fig 3(a)–3(c) display the stable soliton for *W*_1_ = *W*_2_ = 0.25 while Fig 3(d)–3(f) display the unstable soliton for *W*_1_ = *W*_2_ = 0.55; for the fixed parameters *a*_1_ = *a*_2_ = −1, *A*_1_ = 0.5, *V*_1_ = *V*_2_ = 8, Fig 4(a)–4(c) display the stable soliton for *W*_1_ = *W*_2_ = 3 while Fig 4(d)–4(f) display the unstable soliton for *W*_1_ = *W*_2_ = 2.

**Fig 5:**
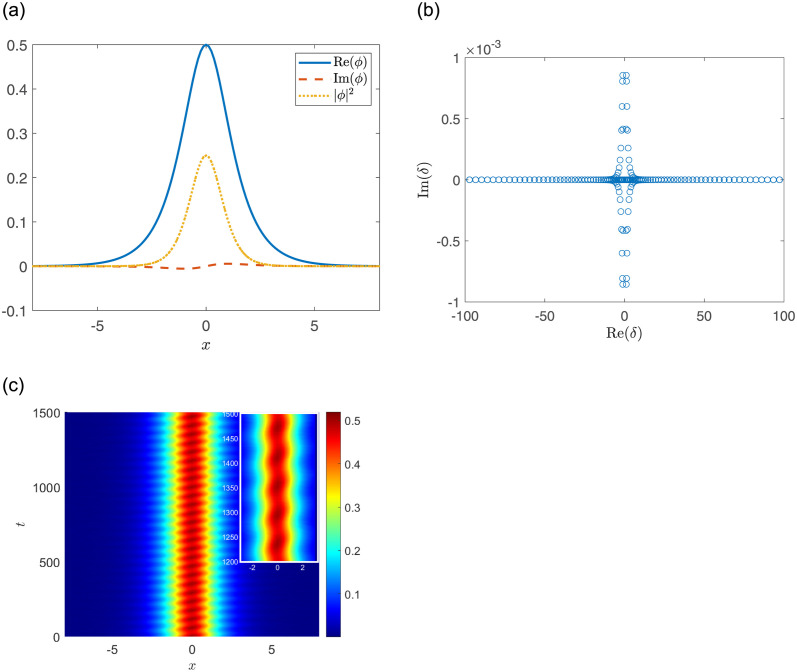
(a) The soliton solutions. (b) Linear stability eigenvalues. (c) Stable propagations of nonlinear modes. The parameters are chosen as: *a*_1_ = 1, *A*_1_ = 0.5, *V*_1_ = *V*_2_ = 0.01, *W*_1_ = *W*_2_ = 0.06.

Furthermore, in the focusing case, the amplitude of the nonlinear mode is periodically oscillating when *V*_1_ and *W*_1_ are sufficiently small, and it experiences more than 5 periods within 1200 ≤ *t* ≤ 1500 [see Fig 5].

## 4 Adiabatic excitation for the nonlinear modes

**Fig 6:**
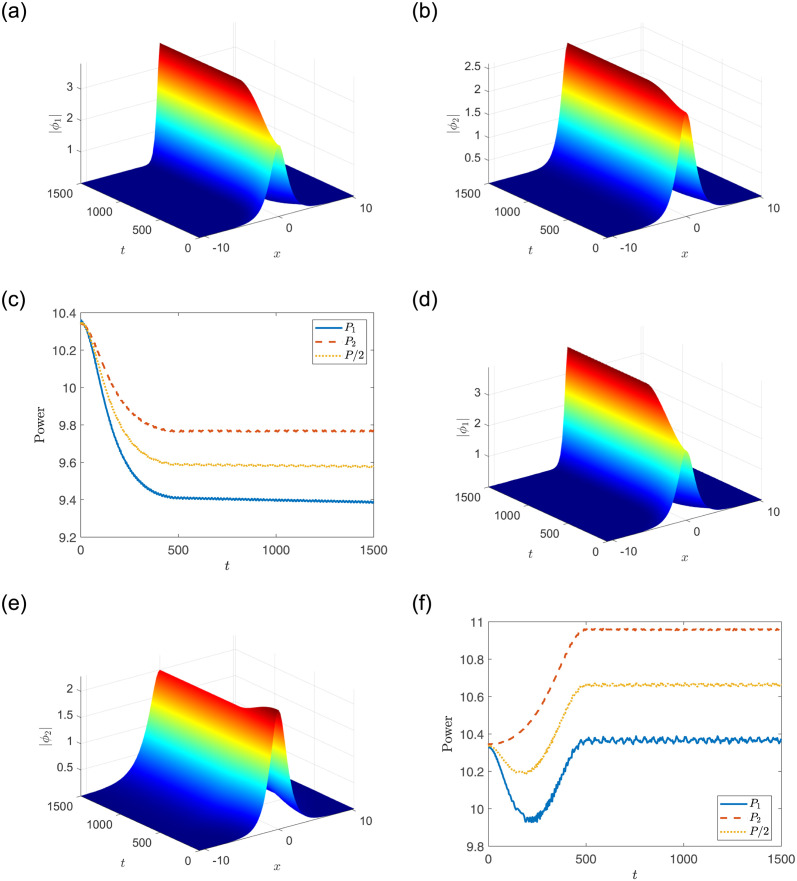
Adiabatic excitation of nonlinear mode and its evolution. The parameters are chosen as: *A*_1_ = *A*_2_ = 2.2733, *W*_1_ = *W*_2_ = 0.55, $V_{1}^{\left(\text{ini}\right)}=1$, $V_{1}^{\left(\text{end}\right)}=2$, $a_{1}^{\left(\text{ini}\right)}=0.1$, $a_{1}^{\left(\text{end}\right)}=1$ and (a-c) $V_{2}^{\left(\text{ini}\right)}=1$, $V_{2}^{\left(\text{end}\right)}=2$, *a*_2_ = 0.1; (d-f) $V_{2}^{\left(\text{ini}\right)}=2$, $V_{2}^{\left(\text{end}\right)}=1$, *a*_2_ = 0.0033.

In this section, we consider excitations of the above-mentioned solitons via adiabatical changes of system parameters. We change the parameters as the functions of *t*. To modulate the system parameters smoothly, we consider the following “switch-on” function:

$$\begin{array}{c} \zeta \left(t\right)=\{\begin{array}{cc} \zeta^{\left(\text{ini}\right)}, & t=0,\\ \frac{\zeta^{\left(\text{end}\right)}-\zeta^{\left(\text{ini}\right)}}{2}[1+\;\text{sin}(\frac{\pi t}{500}-\frac{\pi }{2})]+\zeta^{\left(\text{ini}\right)}, & 0<t<500,\\ \zeta^{\left(\text{end}\right)}, & 500\leq t\leq 1500, \end{array} \end{array}$$ (13)

where *ζ*^(ini)^, *ζ*^(end)^ respectively represent the real initial-state and final-state parameters [38, 45, 54]. Adiabatic excitation includes two stages: excitation stage (0 < *t* < 500) and propagation stage (500 ≤ *t* ≤ 1500). We consider two cases of excitations by setting *a*_1_, *V*_1_ and *V*_2_ to be functions of *t*, that is *a*_1_ → *a*_1_(*t*), *V*_1_ → *V*_1_(*t*) and *V*_2_ → *V*_2_(*t*). To facilitate the display of power changes over time, we set *A*_1_ = *A*_2_, *W*_1_ = *W*_2_ = 0.55, $V_{1}^{\left(\text{ini}\right)}=1$, $V_{1}^{\left(\text{end}\right)}=2$, $a_{1}^{\left(\text{ini}\right)}=0.1$, $a_{1}^{\left(\text{end}\right)}=1$. Firstly, we set $V_{2}^{\left(\text{ini}\right)}=1$, $V_{2}^{\left(\text{end}\right)}=2$, *a*_2_ = 0.1, and the power of nonlinear modes is reduced [see Fig 6a–6c]. Then, we set $V_{2}^{\left(\text{ini}\right)}=2$, $V_{2}^{\left(\text{end}\right)}=1$, *a*_2_ = 0.0033, and the total power of nonlinear modes is to decrease and then increase [see Fig 6d–6f]. The above results mean that the power is not conserved during adiabatic excitations, and it has a correlation with the initial and final state potential parameters.

## 5 Conclusion

In conclusion, we study the nonlinear modes in two-component Bose-Einstein condensates with PT-symmetric Scarf-II potential, which can be described by the coupled Gross-Pitaevskii equations. We investigate the linear stability of the nonlinear modes and validate the results by evolving them with 5% perturbations as an initial condition. We find that solitons tend to be unstable with the increase of |*W*_1_| in the focusing case. It is worth noting that when |*W*_1_| = 3, solitons are stable in the defocusing case. In the focusing case, the amplitude of the nonlinear mode is periodically oscillating when *V*_1_ and *W*_1_ are sufficiently small. Finally, we consider excitations of the solitons via adiabatical changes of system parameters, then we find that the power is not conserved during this adiabatic excitation. These findings of nonlinear modes can be potentially applied to physical experiments of matter waves in Bose-Einstein condensates.

In addition, we can consider other PT-symmetric potentials in the coupled Gross-Pitaevskii equations. Due to the limitations of the parameters in this model, the amplitudes of the two solutions are constrained by a certain relationship. Therefore, we can also consider the case of unequal intra-component and inter-component interactions.
